# Gene expression profiling signatures for the diagnosis and prevention of oral cavity carcinogenesis-genome-wide analysis using RNA-seq technology

**DOI:** 10.18632/oncotarget.4420

**Published:** 2015-06-10

**Authors:** Xiao-Han Tang, Alison M. Urvalek, Kwame Osei-Sarfo, Tuo Zhang, Theresa Scognamiglio, Lorraine J. Gudas

**Affiliations:** ^1^ Department of Pharmacology, Weill Cornell Medical College, New York, NY, USA; ^2^ Genomics Resources Core Facility, Weill Cornell Medical College, New York, NY, USA; ^3^ Department of Pathology, Weill Cornell Medical College, New York, NY, USA

**Keywords:** cancer diagnosis, squamous cell carcinoma, tongue lesions, RNA-seq

## Abstract

We compared the changes in global gene expression between an early stage (the termination of the carcinogen treatment and prior to the appearance of frank tumors) and a late stage (frank squamous cell carcinoma (SCC)) of tongue carcinogenesis induced by the carcinogen 4-nitroquinoline 1-oxide (4-NQO) in a mouse model of human oral cavity and esophageal squamous cell carcinoma. Gene ontology and pathway analyses show that increases in “cell cycle progression” and “degradation of basement membrane and ECM pathways” are early events during SCC carcinogenesis and that changes in these pathways are even greater in the actual tumors. Myc, NFκB complex (NFKB1/RELA), and FOS transcription networks are the major transcriptional networks induced in early stage tongue carcinogenesis. Decreases in metabolism pathways, such as in “tricarboxylic acid cycle” and “oxidative phosphorylation”, occurred only in the squamous cell carcinomas and not in the early stages of carcinogenesis. We detected increases in ALDH1A3, PTGS2, and KRT1 transcripts in both the early and late stages of carcinogenesis. The identification of the transcripts and pathways that change at an early stage of carcinogenesis provides potentially useful information for early diagnosis and for prevention strategies for human tongue squamous cell carcinomas.

## INTRODUCTION

Oral cavity squamous cell carcinomas (OCSCCs), including tongue cancers, account for approximately one half of all head and neck squamous cell carcinomas (HNSCCs) [1]. The 5-year survival rate for patients with OCSCC has not significantly changed despite various treatment improvements in the last several decades [2]. Two major etiological factors in OCSCC are tobacco and alcohol [3, 4]. About 60-70% of oral cavity carcinoma cases are diagnosed only after the tumors have become locally advanced [4]. Currently, the most common approach for assessing the malignancy potential of oral lesions involves histopathological diagnoses of epithelial dysplasia grades that are classified as none, mild, moderate, and severe [5]. Because of the high morbidity and mortality of advanced stage OCSCC, it is important to identify biomarkers that could be used in addition to histopathological assessment for predicting malignancy. These biomarkers could also be very useful in human populations with a high risk for oral cancer, such as people who smoke and drink heavily.

Molecular biological markers have been reported to be useful in assessing cancer risk, distinguishing human oral leukoplakia subtypes (low and high grade dysplasia), and assessing OCSCCs using genome-wide comparison analysis [5-7]. Additionally, because the patient samples analyzed in these studies [5-7] were obtained from a variety of sites in the human oral cavity, including the tongue, palate, lower/upper gingiva, floor of mouth, buccal mucosa, and sinus, the results may not reflect the gene expression profiles at specific sites. These studies [5-7] used cDNA microarrays that have disadvantages when compared with RNA-seq technology. Microarrays have higher background noise, limited specificity and dynamic range for quantifying gene expression levels, and lack of the ability to distinguish different isoforms [8]. Moreover, no data have been reported on the genome-wide gene expression changes in people at a high risk of developing oral cancer, such as people who smoke and drink heavily.

We generated a mouse model of oral SCC by adding the carcinogen 4-nitroquinoline 1-oxide (4-NQO) to drinking water; this model shows major similarities to human oral cavity SCCs in terms of its morphological, histopathological, and molecular characteristics [9-11]. As described in our previous studies [9-11], mouse tongues do not show obvious oral cavity lesions at the termination of the 4-NQO treatment and frank oral SCCs develop in the weeks of post 4-NQO treatment. Thus, this 4-NQO model can also be used to mimic the early stages of tongue carcinogenesis in people who do not yet show obvious oral lesions. We conducted RNA-seq analyses to compare the transcript levels in mouse tongues at the termination of the 4-NQO treatment, at a point when no visible tumors are present, with those in tongue tumors induced by the 4-NQO treatment. The results from this comparison can provide new information useful for the early diagnosis of human oral cancers.

## RESULTS

### Gene expression profiles were altered in the 4-NQO treated tongues before the appearance of tumors

**Figure 1:**
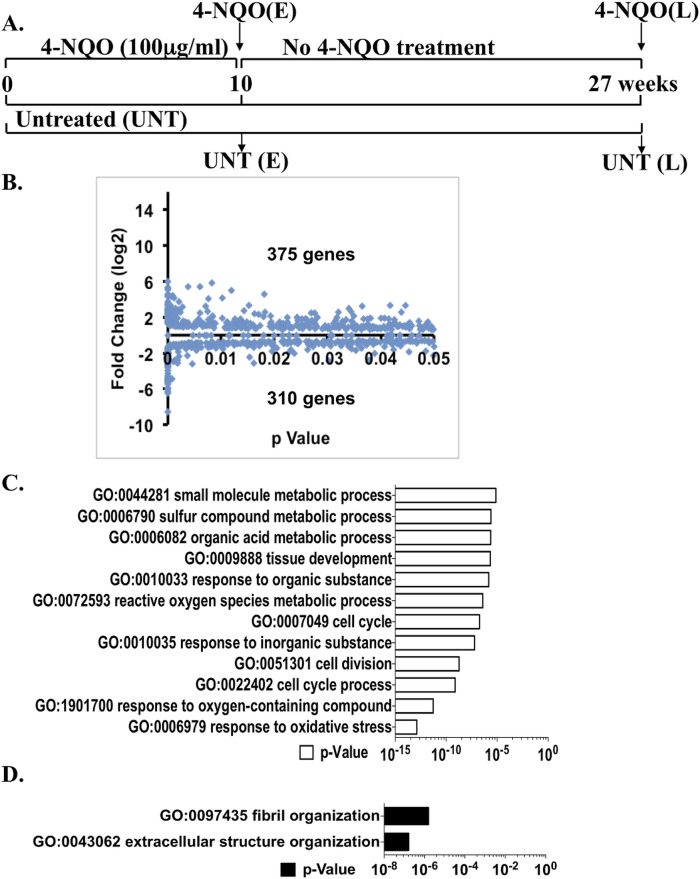
Global changes in transcript levels by RNA-seq and gene ontology (GO) analyses of the genes that are significantly different (*p* < 0.05) between the 4-NQO(E) and the UNT(E) group **A.** Diagram of the experimental protocol (see MATERIALS AND METHODS). **B.** Total numbers of genes with statistically significant increases or decreases (*p* < 0.05) between the 4-NQO(E) group and the UNT(E) group. The mRNA samples extracted from tongues at the termination of the 4-NQO treatment were subjected to RNA-seq analysis. **C.** GO analysis on genes whose mRNA levels were significantly up-regulated in the 4-NQO(E) group compared to the UNT(E) group. **D.** GO analysis on genes whose mRNA levels were significantly down-regulated in the 4-NQO(E) group compared to the UNT(E) group. 4-NQO(E) and UNT(E), the 4-NQO treated tongue group and untreated tongues, respectively, at the time point of termination of the 4-NQO treatment.

All of the mice survived the 10 week 4-NQO treatment. Total RNA from entire mouse tongues treated with 4-NQO or vehicle (no 4-NQO) for 10 weeks was extracted and subjected to RNA-seq analysis (Figure 1A). This total expression analysis generated a set of a total of 23,236 transcripts. A gene was regarded as transcribed if the lower boundary of the confidence interval and the FPKM were greater than zero. We compared the gene expression profiles from the tongues not treated with 4-NQO (early untreated, or UNT(E)) and the 10-week 4-NQO treated tongues (4-NQO(E)) (Figure 1A). Out of the annotated 23,236 transcripts, we discovered 685 genes whose transcript levels were altered significantly (*p* < 0.05) by the 4-NQO treatment, including 375 over-expressed genes and 310 under-expressed genes (Figure 1B, Table S1).

### Gene ontology (GO) analysis

We then conducted a gene ontology (GO) enrichment analysis on a web GO analysis tool ConsensusPathDB [12] using level 3 of the “Biological Process” domain (*p* < 0.00001). GO analysis revealed that compared to the UNT(E) group, the differentially over-expressed gene expression profile in the 4-NQO(E) group favored cell mitosis, and the top categories with obvious over-representation were “cell cycle process” and “response to oxidative stress.” The categories that were under-represented were “extracellular structure organization” (Figure 1C, 1D). Based on these results, the 4-NQO treated tongue epithelial cells were hypothesized to have a stronger tendency to proliferate and migrate than untreated tongue epithelial cells.

### Pathway analysis

In order to understand which pathways were affected by the 4-NQO treatment, we performed a pathway enrichment analysis on the genes whose expression was altered in the 4-NQO(E) group by using the ConsensusPathDB web tool. Pathway based analysis (*p* < 0.00001) revealed that among the top pathways (*i.e.* the p value is small) out of 23 over-represented pathways, “glutathione metabolism”, “metabolism of xenobiotics by cytochrome P450”, and “biological oxidations” pathways showed higher transcript levels, and “mitotic M-M/G1 phases” pathway up-regulation indicated a potential increase in cell proliferation (Table S2). The top pathways among the 9 under-represented pathways included “protein digestion and absorption”, “collagen biosynthesis and modifying enzymes”, “ECM-receptor interaction”, and “focal adhesion” (Table S2), suggesting an increase in cell motility because collagens are very important components of basement membrane and the ECM-receptor interaction limits cell motility [13].

### Induced transcription network module analysis

**Figure 2:**
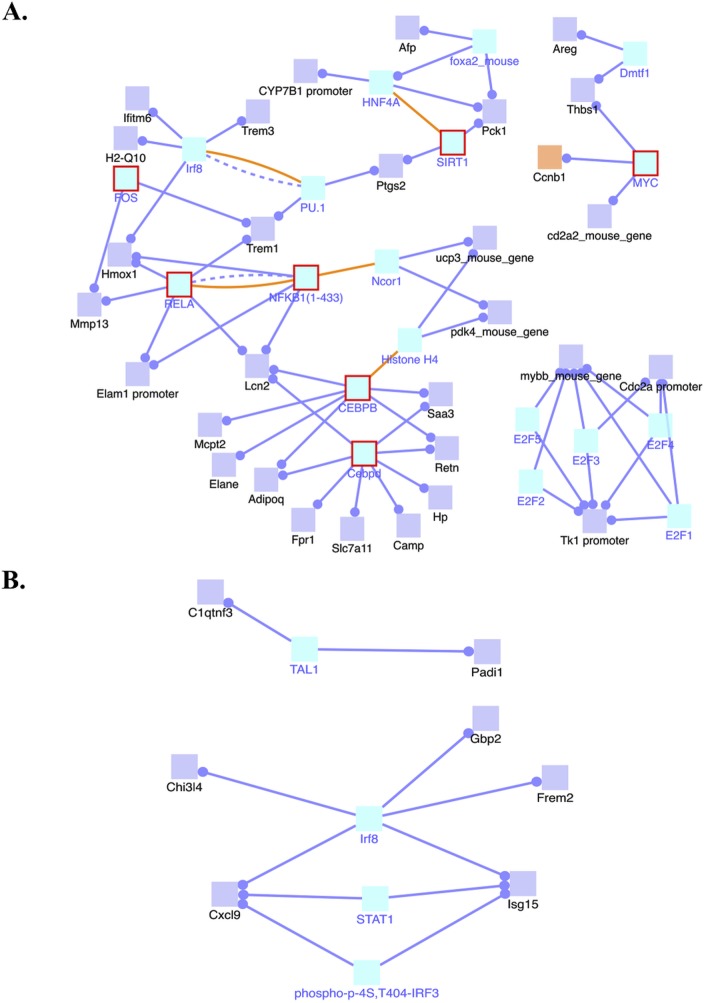
Induced transcription network modules derived from the RNA-seq data in the 4-NQO(E) group compared to the UNT(E) group **A.** Induced transcription networks that contribute to the up-regulations of gene transcripts in the 4-NQO(E) group compared to the UNT(E) group. **B.** Transcription networks that contribute to the down-regulation of gene transcripts in the 4-NQO(E) group compared to the UNT(E) group. Blue lines, transcriptional regulation; yellow lines, protein interactions. 4-NQO(E) and UNT(E), the 4-NQO treated tongue group and untreated tongues, respectively, at the time point of termination of the 4-NQO treatment.

Because we observed changes in global gene expression in the 4-NQO(E) group compared to the UNT(E) group we conducted an induced transcription network module analysis that indicates the interactions of genes whose transcripts were altered in the 4-NQO treated tongues using the ConsensusPathDB web tool [14]. This analysis can reveal functional or physical relationships between the genes and provide information on why these genes are identified together by RNA-seq. These networks are deduced by a *z*-score calculated for each intermediate node that is not in the input gene list with the binomial proportions test [14]. We focused on gene regulation in this study, and analysis of transcripts significantly increased in the 4-NQO(E) group revealed that several transcription network modules were induced, including the Myc transcription network that is associated with activation of the transcription of Ccnb1 (cyclin B1), the NFκB complex (NFKB1/RELA) transcription network, the FOS transcription network that is associated with increases in the MMP13 transcript level, and the SIRT1 transcription network that is associated with activation of PTGS2 gene transcription (Figure 2A).

Our analysis of the mRNAs whose levels were significantly decreased in the 4-NQO(E) group showed that T-cell acute lymphocytic leukemia 1 (TAL1), interferon regulatory factor 8 (IRF8), and the STAT1 transcription network modules were suppressed network modules (Figure 2B). These results indicate that the 4-NQO treatment changed genome-wide RNA expression profiles by modulating several important transcription networks.

### Analysis of gene expression profiles altered in the 4-NQO-induced tongue tumors

**Figure 3:**
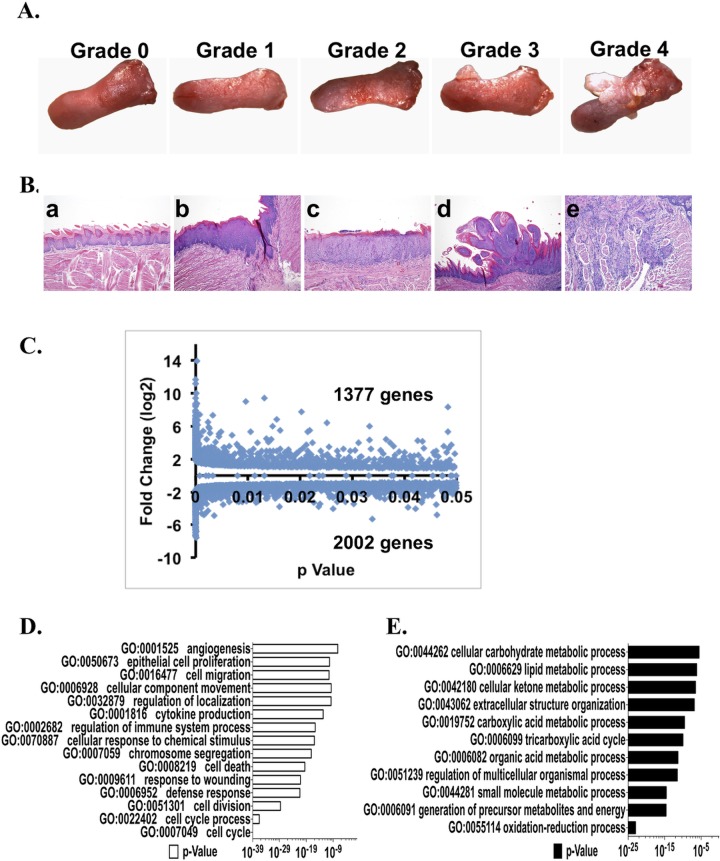
Tongue tumor development, global changes in transcript levels detected by RNA-seq, and gene ontology (GO) analyses of the genes that are different (*p* < 0.05) between the 4-NQO(L) group and the UNT(L) group **A.** Representative gross morphology of tongues from mice in this study and the gross tongue lesion grading system (8×), severity 4>3>2>1>0. **B.** Representative pathological stages of tongue lesions: a, normal (untreated tongue); b, hyperplasia; c, dysplasia; d, papilloma; e, invasive squamous cell carcinoma. **C**. Total numbers of genes with statistically significant increases or decreases (*p* < 0.05) between the 4-NQO(L) group and the UNT(L) group. **D.** GO analysis of genes whose mRNA levels were up-regulated by ≥ 2 fold in the 4-NQO(L) compared to the UNT(L) group. **E.** GO analysis on genes whose mRNA levels were down-regulated by 25% in the 4-NQO(L) group compared to the UNT(L) group. 4-NQO(L) and UNT(L), the 4-NQO induced tongue tumors and untreated tongues, respectively, at the time point of 17 weeks post termination of the 4-NQO treatment.

Multifocal, precancerous and cancerous lesions (papillomas, squamous cell carcinomas) developed during the 17 week, post-4-NQO treatment period in all 4-NQO treated mice (Figure 3A) and pathological analyses showed that these lesions ranged from hyperplasias to malignant squamous cell carcinomas (Figure 3B), consistent with our previous findings [9, 10, 15-17]. Therefore, in addition to the analyses of the alterations in gene expression profiles in the 4-NQO(E) samples, we performed RNA-seq to measure global gene expression changes in in the late untreated (UNT(L)) samples *vs*. the late 4-NQO treated (4-NQO(L)) samples. As we reported previously [17], the mRNA levels of a total of 3379 genes were over-expressed or under-expressed (*p* < 0.05) in the late untreated (UNT(L)) samples *vs*. the late 4-NQO treated (4-NQO(L)) samples (Table S3). Gene ontology (GO) analysis showed that in the 4-NQO induced tongue tumors, categories related to cell mitosis and cell migration were over-represented; the under-expressed GO categories indicated a reduction in ATP production from tricarboxylic acid (TCA) cycle and oxidative phosphorylation in the tongue tumors (Figure 3C and 3D), and an environment for cell migration in the tumors [17]. Moreover, pathway analysis gave similar results (Table S4) [17].

In some pathways that were altered in both the 4-NQO(E) group and the 4-NQO(L) group, the numbers of genes whose mRNA levels were significantly altered in the 4-NQO (L) group were greater than in the 4-NQO(E) group, such as the pathway “Cell Cycle, Mitotic” in which the number of the affected genes in the 4-NQO(L) group was 93, compared to 23 in the 4-NQO(E) group. These data suggest that as tumors form the transcript levels of additional genes are changed, and the changes in transcripts in the 4-NQO induced tumors are broad and reflect the characteristics of these cells, such as enhanced cell proliferation and mobility and abnormal metabolism.

### Induced transcription network module analysis

**Figure 4:**
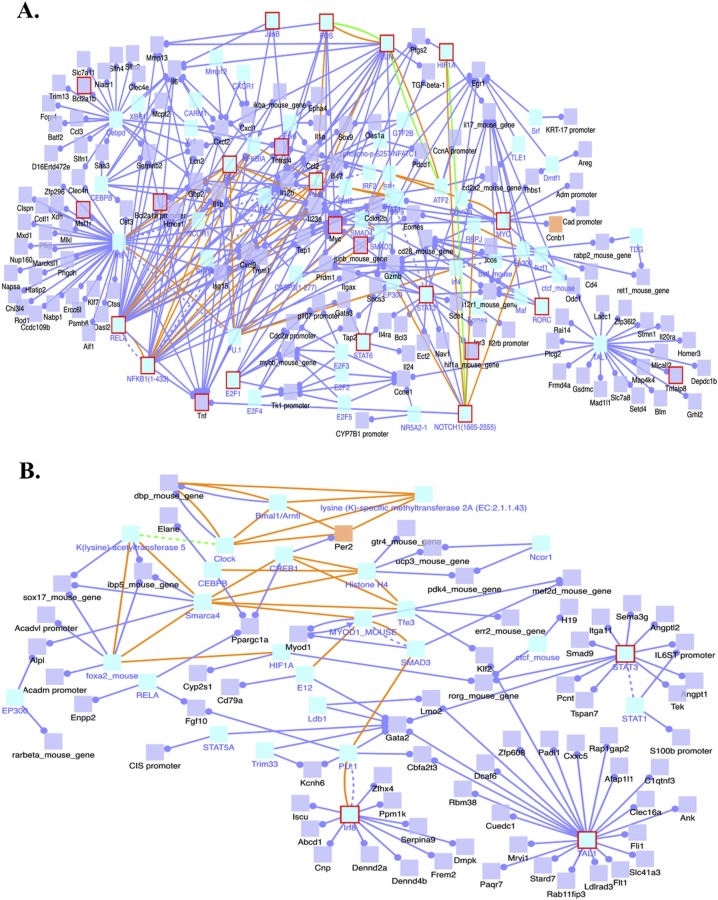
Induced transcription network modules derived from the RNA-Seq data in the 4-NQO(L) group compared to the UNT(L) group **A.** Induced transcription networks that contribute to the up-regulation of gene transcripts in the 4-NQO(L) group compared to the UNT(L) group. **B.** Transcription networks that contribute to the down-regulation of gene transcripts in the 4-NQO(L) group compared to the UNT(L) group. Blue lines, transcriptional regulation; yellow lines, protein interactions. 4-NQO(L) and UNT(L), the 4-NQO induced tongue tumors and untreated tongues, respectively, at a time point of 17 weeks post termination of the 4-NQO treatment.

Our analysis of induced transcription network modules of the genes whose transcripts were increased in the 4-NQO(L) group revealed the activation of several transcription network modules of Myc, NFκB complex (NFKB1/RELA), FOS, JUN, STAT3, STAT6, and Notch1. Among them, we observed induction of the Myc, NFκB complex (NFKB1/RELA), and FOS transcription networks in the 4-NQO(E) group (Figure 4A).

The genes whose transcript levels were lower in the 4-NQO(L) group than in the UNT(L) group showed deactivation of several transcription networks, including that of STAT3, with different target genes (Figure 4B). These data show that as tumor cells proliferate, more transcription networks are modulated that can facilitate tumor development.

### Comparison of transcripts in the 4-NQO induced murine tongue tumors and human head and neck tumors

We also used Oncomine, an online high-throughput cancer biology database and analysis platform, to investigate the relevance of genes altered in 4-NQO induced tumors to human tongue OCSCCs. Table S5 shows that compared to normal human tissues, some mRNAs that were increased or decreased in the 4-NQO(L) *vs*. the UNT(L) group were also differentially expressed in human tongue cancers *vs*. normal tissues, e.g. HIF1α, GLUT1, PTGS2 (Cox-2), cyclin A2, B1, B2, E1, CDK1 and 6, MMP 3, 9, 10, 12, 13, and 14, PDHA1, and Ndufa1. Moreover, gene ontology (GO) (Figure S1) and pathway analyses (Table S6) of a human head and neck cancer RNA-seq database from the Cancer Genome Atlas Data Portal (https://tcga-data.nci.nih.gov/tcga/) revealed that the cell cycle pathway, the citric acid (TCA) cycle, and the respiratory electron transport chain were the most important pathways differentiating normal from human head and neck cancer samples. These data demonstrate that the 4-NQO induced mouse tongue tumors mimic human head and neck cancers, including tongue cancers, not only morphologically and histopathologically, but also at the mRNA level.

### Comparison of gene expression profiles in the 4-NQO(E) and 4-NQO(L) groups

#### Heatmap analyses

We selected some of the genes in cancer-related pathways that were changed in the 4NQO(L) group (compared to the UNT(L) group) for heatmap analyses. The increases in the transcript levels of many genes involved in cell cycle progression and mitosis pathways in the 4NQO(L) group (compared to the UNT(L) group) were greater than those in the 4-NQO(E) group (compared to the UNT(E) group) (Figure S2). Many genes involved in ECM breakdown and cell migration, such as MMP3, 9, 10, 12, and 14, whose transcript levels were greatly enhanced in the 4-NQO(L) group (compared to the UNT(L) group), did not differ between the UNT(E) group and the 4-NQO(E) group. However, matrix metalloproteinase (MMP) 13 transcripts were increased in both the 4-NQO(E) and the 4-NQO(L) groups (compared to the UNT groups). We detected reductions in the transcript levels of many ECM components, such as collagen 6a1, 6a2, and 8a2 (COL6a1, a2, 8a2), in the 4NQO(E) group (compared to the UNT(E) group), and these reductions were greater in the 4-NQO(L) group (compared to the UNT(L) group) (Figure S3). These data suggest that the changes in the mRNA levels of cell cycle regulators and some ECM components are early events that eventually lead to the tumors induced by 4-NQO.

We found increases in transcripts of genes in the HIF1α pathway, i.e. HIF1α; slc2a1 (GLUT1); and slc2a3 (MCT4), only in the 4-NQO(L) group (compared to the UNT(L) group), and not in the 4-NQO(E) group (compared to the UNT(E) group). The decreases in many of the transcripts involved in the tricarboxylic acid (TCA) cycle and oxidative phosphorylation (OX-PHOS) were much greater in the 4-NQO(L) group than in the 4-NQO(E) group, relative to the UNT(L) and UNT(E) groups, respectively (Figure S4). These data indicate that the levels of transcripts involved in energy metabolism are altered only in later stages of oral carcinogenesis induced by 4-NQO.

### Comparison of mRNA levels of individual genes that play important roles in the pathways described above

**Figure 5:**
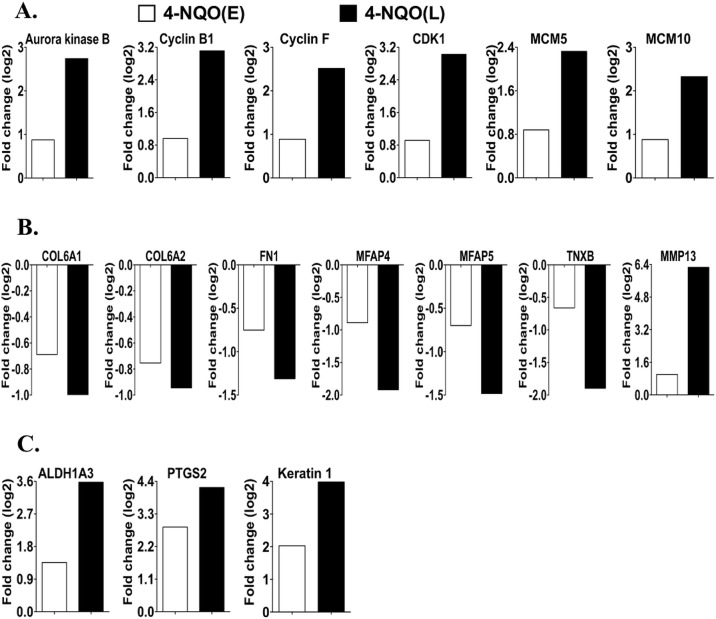
Comparison of the transcript levels of genes involved in cell proliferation, basement membrane and extracellular matrix (ECM) degradation, and oral cancer at the early and late stages of tongue carcinogenesis **A.** Genes involved in cell cycle regulation and DNA replication. The gene IDs are 20877 for aurora kinase B, 268697 for cyclin B1,12449 for cyclin F, 12534 for CDK1, 17218 for MCM5, and 70024 for MCM10. **B.** Genes involved in the ECM breakdown and cell migration. The gene IDs are 12833 for Col6A1, 12834 for Col6A2, 14268 for Fn1, 79263 for Mfap4, 50530 for Mfap5, 81877 for Tnxb, and 17386 for MMP13. **C.** Human oral cancer markers. The gene IDs are 56847 for Aldh1a3, 19225 for Ptgs2, 16678 for keratin 1. COL, collagen; FN1, fibronectin; MCM, minichromosome maintenance complex; MFAP, microfibrillar-associated protein; PTGS2, prostaglandin-endoperoxide synthase 2; and TNXB, tenascin XB. 4-NQO(E), 4-NQO treated tongue group at the time point of termination of the 4-NQO treatment. 4-NQO(L), 4-NQO induced tongue tumors at a time point of 17 weeks post termination of the 4-NQO treatment.

As described above, the 4-NQO treatment affected many GO categories and pathways. We then compared the changes in the mRNA levels of some individual genes that are important in the GO categories and pathways described above between the 4-NQO(E) group and the 4-NQO(L) group (compared to their UNT groups). In the 4-NQO(E) group transcripts involved in cell cycle progression and DNA synthesis, including aurora kinase B (1.8 fold); CDK1 (1.9 fold); cyclin B1 (2.0 fold); and cyclin F (1.9 fold) were increased [18, 19]. Minichromosome maintenance (MCM) complex members 5 (1.8 fold) and 10 (2.4 fold) that participate in unwinding double stranded DNA at origins, recruiting DNA polymerases, and initiating DNA synthesis [20] were also increased. The 4-NQO(L) group showed greater increases in these transcripts (Figure 5A) (aurora kinase B (6.8 fold); CDK1 (8.3 fold); cyclin B1 (8.8 fold); cyclin F (5.8 fold); MCM 5 (5.1 fold); and MCM 10 (8.0 fold)). Additional transcripts involved in cell cycle progression and DNA synthesis, including aurora kinase A (4.9 fold); CDK6 (6.1 fold); cyclin A2 (4.6 fold), B2 (5.7 fold), and E1 (7.7 fold) [18, 19]; minichromosome maintenance (MCM) complex members 2 (2.6 fold), 3 (2.9 fold), 4 (3.0 fold), 6 (2.3 fold), and 7 (2.7 fold); DNA replication helicase 2 (DNA2) (2.9 fold); DNA ligase 1 (LIG1) (3.1 fold); origin recognition complex, subunit 1 (ORC1) (7.2 fold); DNA polymerase, alpha 1 (POLA1) (2.2 fold); and DNA primase large subunit (PRIM2) (2.7 fold), increased in the 4-NQO(L) group as compared to the 4-NQO(E) group. Thus, some members of the cell cycle regulatory network are activated during the early stages of tongue carcinogenesis, before the appearance of visible tumors, and additional members of the cell cycle regulatory network are transcriptionally activated in the 4-NQO induced tumors.

Normal epithelial cells are anchored three-dimensionally through interactions with the ECM. The basement membrane provides structural support for epithelial cells, and epithelial cells establish connecting links to the ECM [21]. In malignant tumors the tissue architecture is lost: cells break contacts with their sister cells and migrate through the damaged basement membrane [22]. Transcripts of the basement membrane components collagen (COL) 6a1 (lower by 38%) and 6a2 (lower by 41%) [23]; ECM proteins fibronectin (FN1) (lower by 41%) [24], microfibrillar-associated protein (MFAP) 4 (lower by 46%) [25], and MFAP5 (lower by 38%) [26]; and tenascin XB (TNXB) (lower by 37%) [27] were lower in the 4-NQO(E) than the UNT(E) group. Moreover, transcripts of MMP13, which belongs to a matrix metalloproteinase family that contributes to extracellular matrix (ECM) breakdown and cancer cell migration [28], exhibited greater levels (2.0 fold) in the 4-NQO(E) group than the UNT(E) group. Compared to the decreases observed in the 4-NQO(E) (*vs*. the UNT(E) group), UNT(L) group, transcripts in the 4-NQO(L) group (*vs*. the UNT(L) group) (Figure 5B) that were further decreased included Col6a1 (lower by 50%), Col6a2 (lower by 48%), FN1 (lower by 60%), MFAP4 (lower by 74%), MFAP5 (lower by 64%), and TNXB (lower by 73%). MMP13 mRNA was also further increased (77.6 fold). In addition to these genes, the 4-NQO(L), but not the 4-NQO(E) samples showed increases in the transcript levels of MMPs 3 (7.7 fold), 9 (5.6 fold), 10 (693.1 fold), 12 (30.1 fold), 14 (2.6 fold); tenascin C (TNC), involved in cell migration [29] (4.0 fold); and Slug, which participates in epithelial to mesenchymal transition and cell migration in HNSCC [30] (4.6 fold). These results indicate that the decreases in the production of basement membrane and ECM components are early events (prior to tumor appearance) in OCSCC carcinogenesis and that these decreases persist in the 4-NQO induced tumors.

Moreover, we detected 2.6, 4.1, and 7.3 fold increases in transcripts of aldehyde dehydrogenase 1 family member A3 (Aldh1a3) [31], keratin 1 [32], and prostaglandin-endoperoxide synthase 2 (PTGS2) (also known as cyclooxygenase-2 (COX-2) [4]), in the 4-NQO(E) compared to the UNT(E) group, respectively. The increases in these transcripts were even greater in the 4-NQO(L) group compared to the UNT(L) group: 11.9 fold for Aldh1a3, 15.8 fold for keratin 1, and 18.3 fold for PTGS2 (Figure 5C). Thus, the changes in transcripts for these markers take place early during OCSCC carcinogenesis and these transcripts could potentially be used for the early diagnosis of human oral cancers.

## DISCUSSION

Our comparison by RNA-seq of genome wide gene expression profiles between an early stage of oral cavity carcinogenesis and a later stage in which frank squamous cell carcinomas are present shows that the pathways of “cell cycle progression” and “disruption of ECM and basement membrane” are activated at an early stage of tongue carcinogenesis (Figure 1 and Table S2), and that even greater changes in these pathways occur at late stages. In addition, we show that the transcripts of some cancer markers, such as ALDH1A3 [31] and PTGS2 [4], are increased at both early and late stages of tongue carcinogenesis. In contrast, we detected changes in metabolism pathways, such as the TCA cycle and OX-PHOS, only at late stages of tumorigenesis (Figure 3 and Table S4).

Global transcriptional network information is useful for the interpretation of cancer signatures [33]. Dissection of activated transcriptional programs during carcinogenesis can explain tumorigenic mechanisms and suggest intervention points for the therapy. We identified Myc, NFκB complex (NFKB1/RELA), and FOS transcription networks as major activated transcriptional programs in the 4-NQO(E) group, and these transcription programs remained activated in the 4-NQO(L) group (Figures 2 and 4). Myc is an oncogenic transcription factor in many human cancers [34]. Moreover, c-myc protein is detected in tongue leukoplakia and its level strongly indicates a poor prognosis in human tongue cancer [35]. Compared with normal tissue, a higher level of NFκB protein is positively correlated with progression from leukoplakia to OCSCC [36]. However, the role of FOS in tumor development remains controversial [37]. The combination of a c-myc inhibitor [38] or an inhibitor that suppresses c-myc transcription [39] and an NFκB inhibitor could be an effective approach to inhibit the transcription of cyclin B1, MMP13, and PTGS2, thereby preventing or limiting the progression of carcinogenesis in humans with early stage tongue cancers.

Aurora kinase B protein levels are positively correlated with cancer cell proliferation and tumor progression in human oral squamous cell carcinomas [40]. The activation of the CDK1-cyclin B1 complex leads to mitotic entry [41], and overexpression of cyclin B1 [42] and CDK1 [43] proteins is associated with progression of human oral squamous cell carcinoma. In a recent study, increases in MCM5 protein as human oral cancers (including tongue cancer) progress were reported [44]. In line with these studies, our results show that the increases in these transcripts occur in both the early and late stages of oral carcinogenesis (Figure 5).

It is a remarkable that the transcripts of many genes involved in the biosynthesis of the components of the basement membrane and ECM start to decline in the 4-NQO(E) group prior to the presence of tumors (Figures 1 and 5, Table S2). This interpretation is consistent with the “parallel progression model” of metastasis, and suggests that “quasi-normal” epithelial cells disseminate from pre-neoplastic lesions relatively early during cancer progression [13, 45]. Clinical studies have shown that severe oral leukoplakia with a high risk of malignant transformation shows greater basement membrane disruption than mild oral leukoplakia [46].

ALDH1A3 mRNA and protein levels are greater in advanced stage human HNSCCs than in normal tissue [31], but there have been no reports of the transcript and protein levels of ALDH1A3 in human tongue leukoplakia. The transcript and protein levels of PTGS2 are associated with genomic instability and are involved in the malignant transformation of oral epithelial cells [4]. PTGS2 protein levels in oral leukoplakia are also greater than in the normal tissue [36]. Increases in the transcript and protein levels of keratin 1 have been observed in precancerous lesions of the human oral cavity and OCSCC [32] and in our data [10] (Figure 5).

The identification of these pathways and markers that are altered in the early stage of oral cavity carcinogenesis has several potential uses: 1) to predict the risk of malignant transformation of oral mucosa by using oral brush biopsies, especially in the tongue epithelia of people at a high risk of developing oral cancers but without obvious oral lesions, such as people who smoke and drink heavily; 2) to predict the risk of developing tongue cancers in people who have oral lesions, such as leukoplakias; and 3) to distinguish early and late stages of tongue carcinogenesis by differences in the numbers of genes in altered pathways.

## MATERIALS AND METHODS

### Tumor development in the mouse oral cavity

Six week old wild type C57BL/6 female mice (15 mice/group) were treated with vehicle as a negative control or with 100 μg/ml 4-nitroquinoline-1-oxide (4-NQO) for 10 weeks, as previously described [9, 10]. At this time point tongues from two mice per group were harvested for RNA-seq analysis. The rest of mice were given regular drinking water and subsequently sacrificed at around 17 weeks after the end of the 4-NQO treatment, when tongue tumors had developed; the tongues were then also harvested for RNA-seq analysis. The care and use of animals in this study were approved by the Institutional Animal Care and Use Committee (IACUC) of Weill Cornell Medical College.

### RNA-seq analysis of the mRNA transcriptome

Mouse tongues were snap frozen in liquid nitrogen, and stored at −70°C until total RNA extraction. Total tissue RNA was extracted and subjected to Next-Generation Sequencing (RNA-seq) at the Genomics Resources *Core* Facility, Weill Cornell Medical College (supporting information).

### RNA-seq data analysis

Tophat software was used to align raw sequencing reads to the UCSC mm9 mouse reference genome, and Cufflinks was used to measure transcript abundances in Reads Per Kilobase of exon model per Million mapped reads (RPKM) as well as to find genes with statistically significant changes in expression. The heatmaps for genes of interest were generated by R package.

## SUPPLEMENTARY MATERIAL FIGURES AND TABLES














