# Supplementary material for: Gene expression profiling signatures for the diagnosis and prevention of oral cavity carcinogenesis-genome-wide analysis using RNA-seq technology
Source: Oncotarget. 2015 Jun 10;6(27):24424–35. doi: 10.18632/oncotarget.4420 (PMC4695195; doi:10.18632/oncotarget.4420)
Supplement: Supplementary file 1 [file oncotarget-06-24424-s001.pdf]

Gene expression profiling signatures for the diagnosis and prevention of oral cavity carcinogenesis-genome-wide analysis using RNA-seq technology

Supplementary Material

Tissue dissection, lesion grade measurement, and pathological diagnosis. The tongues of mice were dissected immediately after cervical dislocation. Gross lesions were identified and photographed, and visible cancerous lesions on the tongues were counted for the multiplicity (number of lesions per mouse) examination with a 8× magnification. The severity of gross lesions on the tongues was quantified by a grading system that included 0 (no lesion), 1 (mild lesion), 2 (intermediate lesion), 3 (severe lesion), and 4 (most severe lesion), respectively, and the average grades from different treatment groups were used for the analyses of the tongue lesions. Mouse tongues were cut longitudinally. One part of the tongue was fixed in freshly made 4% paraformaldehyde overnight at 4°C, embedded in paraffin, and sectioned into 7-μm sections. Entire one part of each tongue was immediately snap frozen and stored at -70°C before RNA extraction. The histological diagnosis of squamous neoplasia was performed by a pathologist (T.S.) on the hematoxylin and eosin (H&E) stained, sectioned tissue samples. The lesions observed were classified into three types: epithelial hyperplasia; dysplasia (mild, moderate, and severe); and squamous cell carcinoma (SCC), as described previously [11, 22].

RNA-seq analysis of mRNA transcriptome. Representative tongue samples were chosen for the RNA-seq analysis. The extraction of total cellular RNA from mouse tissues was carried out using the RNeasy kit (Qiagen). Subsequent RNA preparation steps were carried out at the Genomics Resources Core Facility of WCMC. RNA integrity was measured using the Agilent 2100 BioAnalyzer (Agilent Technologies). Samples with RNA integrity number (RIN) values of 10 were used to construct cDNA libraries. mRNA was purified by using pre-prepared Sera-mag Magnetic Oligo(dT) Beads from Illumina Inc. (San Diego, CA), subjected to thermal fragmentation, and reverse transcribed to first

strand cDNA. Following the removal of mRNA strands by RNaseH, first strand cDNAs were used as templates to produce double strand cDNAs, and the overhangs resulting from fragmentation were repaired to blunt ends. An ‘A’ base was added to the 3’ end of cDNAs and subsequently the cDNAs were ligated to Illumina paired end (PE) adaptors that have a single ‘T’ base overhang at their 3' end. The cDNA-adaptor libraries were purified and enriched by 15 cycles of PCR. The enriched libraries were hybridized to a flow cell and amplified, resulting in ultra-high density flow cells with millions of clusters, each containing about 1,000 copies of the templates. The double stranded cDNA-adaptors were denatured and converted into single strand DNA, and then the template cDNAs were amplified one more time isothermally to produce surface-bound colonies. The clonal DNA clusters were linearized, free 3’ OH ends blocked, denatured, hybridized to sequencing primers, and sequenced. The sequencing was conducted by running 4 samples per lane with 51 pair-end cycles on the HISeq2000/1000. The Sequencing-by-Synthesis process used reversible terminators and a DNA polymerase modified to accept reversible terminator nucleotides. After each synthesis cycle the fluorescence of clusters was imaged with high sensitivity. Then the sequencing images were analyzed in three steps, image analysis, base calling, and sequence analysis. The Tophat software was used to align raw sequencing reads against the UCSC mm9 mouse reference genome, and Cufflinks software was employed to measure transcript abundances in the unit of reads Per Kilobase of exon model per Million mapped reads (RPKM), as well as to perform statistical analysis on the changes in gene expression. The heatmaps for genes of interest were generated by R package software.

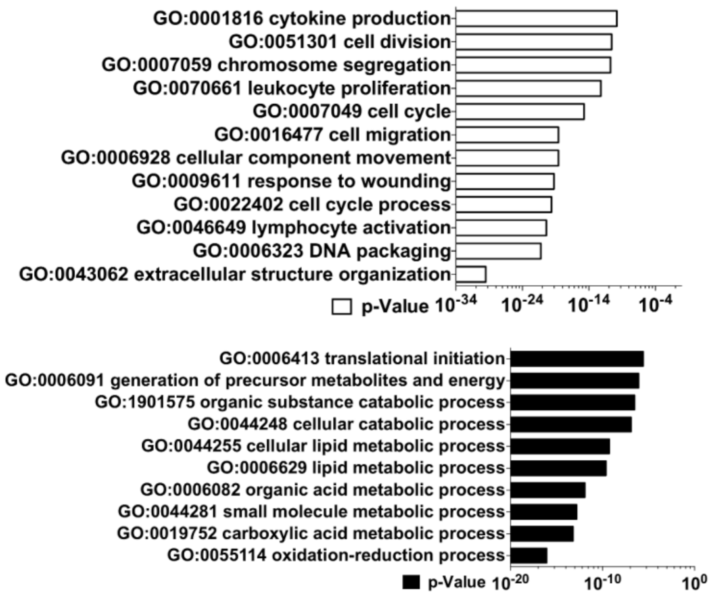

Figure S1: Gene Ontology (GO) analyses of the genes whose transcript levels were different (p<0.001) between human head and neck cancer samples and normal tissues. White bars indicate categories with increases and black bars show categories with decreases in the cancer samples.

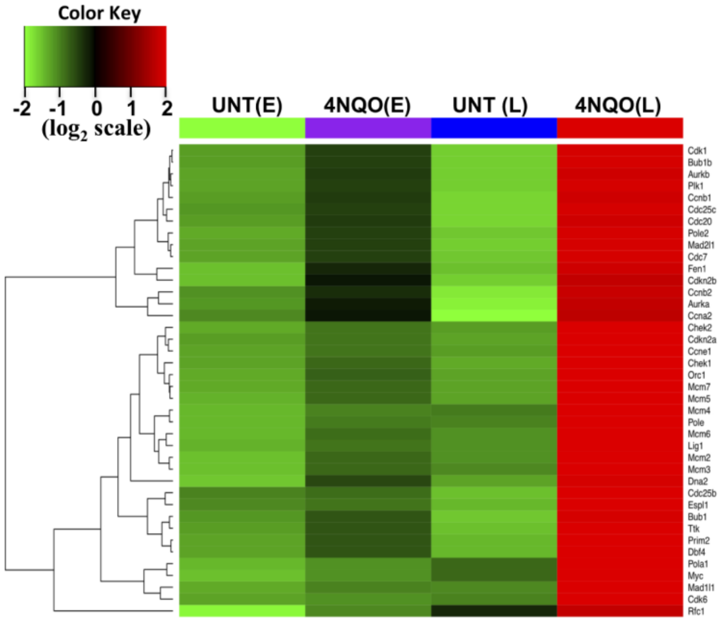

Figure S2: Heatmap analysis on the genes involved in cell proliferation. The RNA–seq data of selected genes involved in cell mitosis and DNA replication were used to generate a heatmap. 4-NQO(E) and UNT(E), the 4-NQO treated tongue group and untreated tongues, respectively, at the time point of termination of the 10 week 4-NQO treatment. 4-NQO(L) and UNT(L), the 4-NQO induced tongue tumors and untreated tongues, respectively, at a time point of 17 weeks post termination of the 4-NQO treatment.

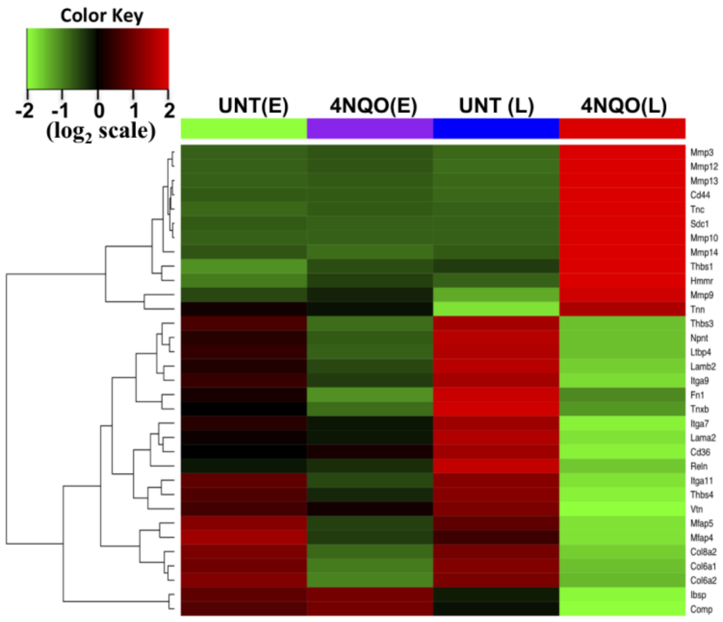

Figure S3: **Heatmap analysis of the genes involved in ECM breakdown and ECM components.** The RNA–seq data of the selected genes involved in ECM breakdown and the selected ECM component genes were used to generate a heatmap. 4-NQO(E) and UNT(E), the 4-NQO treated tongue group and untreated tongues, respectively, at the time point of termination of the 10 week 4-NQO treatment. 4-NQO(L) and UNT(L), the 4-NQO induce tongue tumors and untreated tongues, respectively, at a time point of 17 weeks post termination of the 4-NQO treatment.

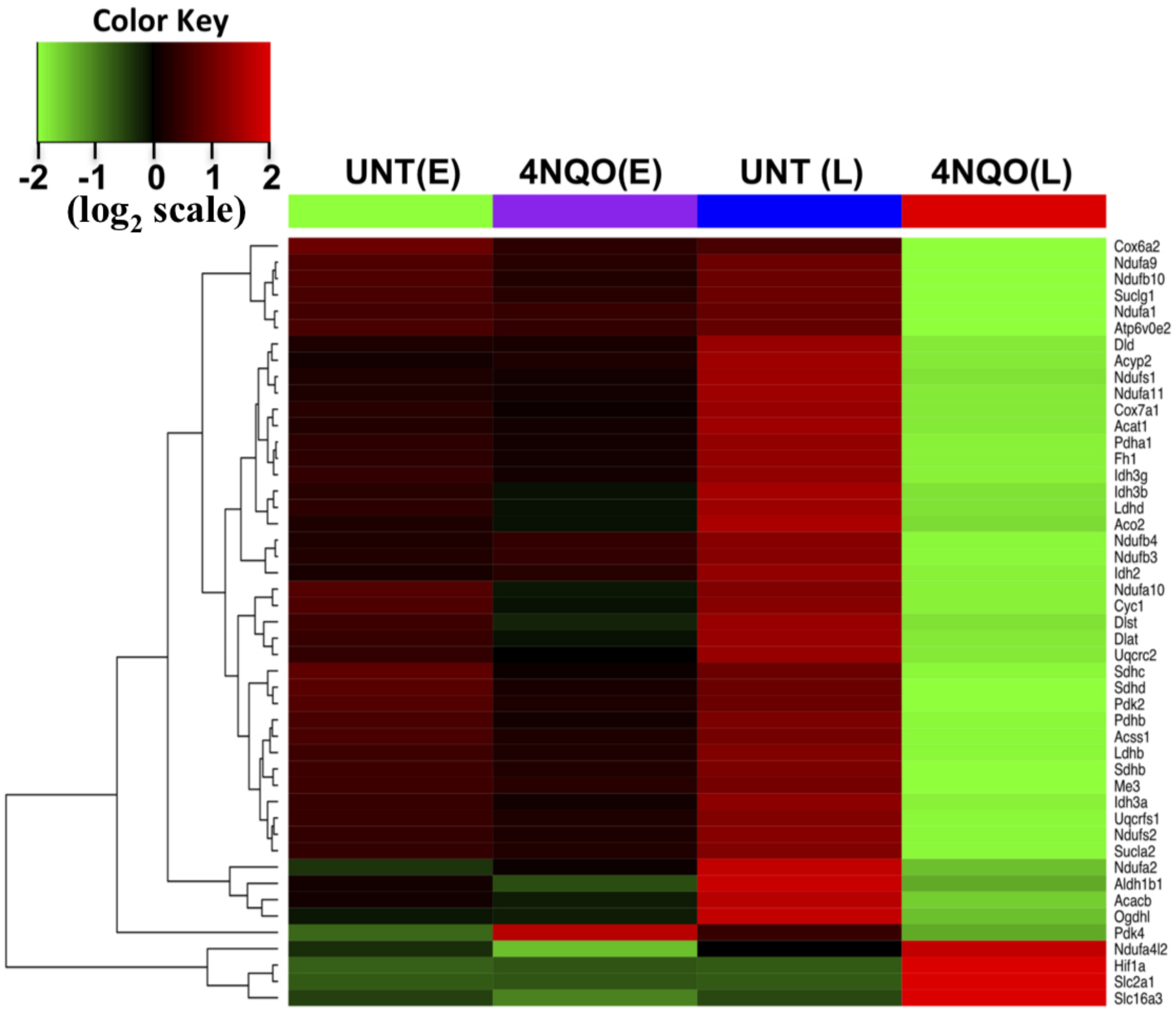

Figure S4: Heatmap analysis of the genes involved in the HIF1α signaling pathway, the TCA cycle, and the OX-PHOS pathway. The RNA–seq data of selected genes involved in the HIF1α signaling pathway, the TCA cycle, and the OX-PHOS pathway were used to generate a heatmap. Slc2a1, GLUT1; Slc16a3, MCT4. 4-NQO(E) and UNT(E), the 4-NQO treated tongue group and untreated tongues, respectively, at the time of termination of the 10 week 4-NQO treatment. 4-NQO(L) and UNT(L), the 4-NQO induced tongue tumors and untreated tongues, respectively, at a time point of 17 weeks post termination of the 4-NQO treatment.
